# Identifying High-Priority Ethical Challenges for Precision Emergency Medicine: Nominal Group Study

**DOI:** 10.2196/68371

**Published:** 2025-02-06

**Authors:** Christian Rose, Emily Shearer, Isabela Woller, Ashley Foster, Nicholas Ashenburg, Ireh Kim, Jennifer Newberry

**Affiliations:** 1 Department of Emergency Medicine, Stanford University School of Medicine, 500 Pasteur Dr, Stanford, CA, 94305, United States, 1 (650) 723-5111; 2 Department of Emergency Medicine, Alpert School of Medicine, Brown University, Providence, RI, United States; 3 Loyola University Chicago, Chicago, IL, United States; 4 Department of Emergency Medicine, University of California, San Francisco, San Francisco, CA, United States

**Keywords:** precision medicine, emergency medicine, ethical considerations, nominal group study, consensus framework

## Abstract

**Background:**

Precision medicine promises to revolutionize health care by providing the right care to the right patient at the right time. However, the emergency department’s unique mandate to treat “anyone, anywhere, anytime” creates critical tensions with precision medicine’s requirements for comprehensive patient data and computational analysis. As emergency departments serve as health care’s safety net and provide a growing proportion of acute care in America, identifying and addressing the ethical challenges of implementing precision medicine in this setting is crucial to prevent exacerbation of existing health care disparities. The rapid advancement of precision medicine technologies makes it imperative to understand these challenges before widespread implementation in emergency care settings.

**Objective:**

This study aimed to identify high priority ethical concerns facing the implementation of precision medicine in the emergency department.

**Methods:**

We conducted a qualitative study using a modified nominal group technique (NGT) with emergency physicians who had previous knowledge of precision medicine concepts. The NGT process consisted of four phases: (1) silent generation of ideas, (2) round-robin sharing of ideas, (3) structured discussion and clarification, and (4) thematic grouping of priorities. Participants represented diverse practice settings (county hospital, community hospital, academic center, and integrated managed care consortium) and subspecialties (education, ethics, pediatrics, diversity, equity, inclusion, and informatics) across various career stages from residents to late-career physicians.

**Results:**

A total of 12 emergency physicians identified 82 initial challenges during individual ideation, which were consolidated to 48 unique challenges after removing duplicates and combining related items. The average participant contributed 6.8 (SD 2.9) challenges. These challenges were organized into a framework with 3 themes: values, privacy, and justice. The framework identified the need to address these themes across 3 time points of the precision medicine process: acquisition of data, actualization in the care setting, and the after effects of its use. This systematic organization revealed interrelated concerns spanning from data collection and bias to implementation challenges and long-term consequences for health care equity.

**Conclusions:**

Our study developed a novel framework that maps critical ethical challenges across 3 domains (values, privacy, and justice) and 3 temporal stages of precision medicine implementation. This framework identifies high-priority areas for future research and policy development, particularly around data representation, privacy protection, and equitable access. Successfully addressing these challenges is essential to realize precision medicine’s potential while preserving emergency medicine’s core mission as health care’s safety net.

## Introduction

Historically, medical treatments have been tailored to the average patient. However, no individual is ever the average patient—everyone has their own risk factors, needs, and ideal outcomes. Precision medicine, as defined by the White House’s nationwide Precision Medicine Initiative, aims to address this problem by moving from a one-size-fits-all model toward one of providing the “right care to the right person at the right time” through the use of vast amounts of health data, including genomic information, electronic health records, environmental and lifestyle data, and medical imaging data, among others [1-5]. However, even the most advanced, targeted therapies are limited by biases that already exist in the US health care system [6,7].

Variation in care, even when “evidence based,” can result in worsened gaps between the haves and the have-nots, disproportionately burdening already marginalized communities [8]. For example, patients with limited resources or inadequate insurance coverage may be unable to access these evidence-based interventions, while well-insured patients at major medical centers can, creating a 2-tiered system where advances in precision medicine may primarily benefit those with better health care access. For emergency physicians, who serve in the safety net for American health care, precision medicine’s “right care, right patient, right time” must be balanced with our “anyone, anywhere, anytime” mantra and the practical and ethical challenges that this setting presents.

Early precision medicine initiatives have focused on the use of genetics to better understand or treat diseases. Much has been made of determining the right medication for the right person (eg, warfarin dosing for those with a CYP [cytochrome P450] mutation) or when to start cancer screening based on genetic profiles (eg, colorectal screening for CHD1 [chromodomain helicase DNA binding protein 1]) [9]. It is clear, however, that genetics alone cannot explain much of the variation in outcomes across the population [2,10]. The effect of behavioral factors like diet and exercise are well-established, and studies have begun to illuminate the effect of social and environmental determinants of health like access to health care resources or housing, which may be responsible for over half of health outcomes [11,12]. As such, there has been a broadening approach to precision medicine that may also include home monitoring, social media and social networks, as well as patient-generated data like diet, daily steps, exercise, or heart rate variability [13,14].

While useful when available, not all patients can afford these tools and services. Furthermore, “precise” treatments rely on the accuracy of this data, which must be built into datasets and shared. Few comprehensive datasets that incorporate all of these data exist, and even fewer have been carefully evaluated for missing or inaccurate data that could bias results [15]. Current datasets suffer from a lack of representation from some populations while simultaneously overrepresenting others. Healthy patients or those without access to care rarely present to hospitals, while those who are chronically ill are seen more often. Furthermore, a skewed representation occurs because research data often comes primarily from large academic centers rather than community hospitals or rural health care settings [16]. Data that were collected where patients are provided differential care might inadvertently support antiquated practices [17-21] due to the association of outcomes with variables like the ability to pay for a higher level of care. Though precision approaches aim to limit quality gaps and provide more equitable care, decisions that are made without the full picture of their context might inadvertently exacerbate systemic biases, for example, when risk assessment scores built from data of principally white cohorts over- or underestimate the risk of disease in non-White populations [7].

The emergency department setting offers additional unique hurdles for the growth of precision medicine. Little patient-generated data are readily available at the point of care. Highly important features like code status are haphazardly available among the sickest patients. Nevertheless, emergency care providers strive to provide better care, and to actualize the benefits of a learning health care system by integrating evidence and experience into continuous improvement and innovation. How can health care’s safety net, responsible for a growing and significant portion of acute care in America [22,23], begin to approach the problems that are inherent to the implementation of precision medicine so that it might offer real-time, equitable care for patients with the widest spectrum of disease in medicine and a microcosm of the health care system?

Thus, the burgeoning expansion of precision medicine underscores the need to identify challenges facing its nascent deployment and ethical use in the emergency setting such that its optimal benefits may be realized. Our main objective was to identify critical ethical challenges in implementing precision medicine in the emergency setting using a consensus technique. This approach would also help identify themes and gaps in current research. This manuscript presents the resulting consensus framework.

## Methods

### Study Design and Setting

We used a modified nominal group technique (NGT), a consensus methodology widely used in emergency medicine, to identify high priority ethical challenges relevant to the implementation of precision medicine in the emergency medicine setting and arrive at a consensus framework [24-31]. This study followed the COREQ (Consolidated Criteria for Reporting Qualitative Research) reporting framework for qualitative studies [32].

**Figure 1.**
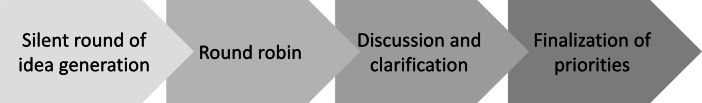
Flow chart of the nominal group technique (NGT) process.

NGT is a 4-step process in which stakeholders generate and then prioritize questions or research ideas (Figure 1) [33]. NGT was well-suited for our research question as our aim was to generate a comprehensive set of ethical challenges which could then be organized and ordered. This is in contrast to other frequently used consensus techniques such as the Delphi technique, which are designed instead to reach a consensus opinion on one particular question [24]. NGT has previously been used successfully to identify research priorities in the field of emergency medicine [25,27,34-37].

### Selection and Participants

A NGT session group size is recommended at greater than 6 participants, with the ideal between 9 and 12 [34]. As precision emergency medicine is an emerging field, we sought participants who had familiarity with the clinical practice of emergency medicine, including an understanding of the types of data that are collected throughout a patient’s journey and the ways in which data is used for clinical decision-making. Participants were recruited through email to the Society of Academic Medicine Ethics Committee listserve; the email invitation also asked recipients for referral and sharing to others who may be interested. A quota sample of the first 14 respondents to the email were registered as potential participants in the NGT group to meet the ideal group size and allow for scheduling conflicts. In total, 12 emergency medicine physicians who reported working knowledge of precision medicine topics, as evidenced by publication records and participation in precision medicine conferences or similar activities, joined our NGT session. Participants represented a spectrum of subspecialties (education, ethics, pediatrics, diversity, equity, inclusion, and informatics) across various stages of training (resident, early-, mid-, and late-career) and practice settings (county hospital, community hospital, academic center, and integrated managed care consortium) (Specialty, Level of Training, and Practice Setting included in Table S1 in Multimedia Appendix 1) to provide a range of emergency medicine practice perspectives.

Participants took part in the NGT session during the American College of Emergency Physicians annual conference in Boston in October 2021. The process was moderated by the lead researcher of the group (CR), and notes were taken by 2 separate researchers (ES and MG).

### NGT Process

#### Phase 1: Silent Idea Generation.

The first step of NGT is a round of silent idea generation. In this phase, after being given an initial prompt, participants silently write down what they believe to be high priority challenges. It is imperative that this phase is silent, as this allows each member of the group to write their initial thoughts without influence from other members. Our prompt asked participants to identify high priority ethical concerns for the implementation of precision medicine initiatives in the emergency medicine setting. They had 10 minutes to complete this phase.

#### Phase 2: Sharing Ideas.

After individual idea generation, participants then shared their ideas with the group in a round-robin (going around the table one person at a time) format. NGT has the advantage of not allowing any one particular voice to outweigh others, by asking each participant to share their complete list of ideas in turn during the round robin phase, rather than ad hoc discussion, thus minimizing potential bias [38,39]. This phase lasted 10 minutes.

#### Phase 3: Discussion and Clarification

After the round-robin format, participants undertook discussion and clarification of topics. In this phase, participants were encouraged to ask other members for clarification of topics generated as needed. Cross-cutting themes were identified, and duplicate topics consolidated. Over 30 minutes, key topics were then placed on moveable cards for prioritization and further thematic grouping.

#### Phase 4: Finalization of Priorities

Finally, participants finalized the topics they believed represented the highest priority areas to address. In traditional NGT, this is the ranking phase, where members are allocated votes to generate a ranked list of priorities. Overwhelmingly, however, participants felt that the topics they raised were interrelated and of commensurate importance over several themes such that their categorization—rather than prioritization—would provide greater impactful focus for interventions. Consequently, participants grouped challenges through consensus opinion over a 40-minute period.

### Ethical Considerations

This study was approved by the Stanford University Institutional Review Board (IRB #62687); participants gave written informed consent before taking part in this study. All data were collected anonymously. Participants received a US $125 gift card in compensation for their time.

## Results

During phases 1 and 2 of the NGT process, 82 challenges were generated (complete list in Table S2 in Multimedia Appendix 1) with the average participant contributing 6.8 (SD 2.9) challenges. After refinement and separation of cross-cutting themes and consolidation of duplicate topics in phase 3, a list of 48 unique challenges remained.

**Figure 2.**
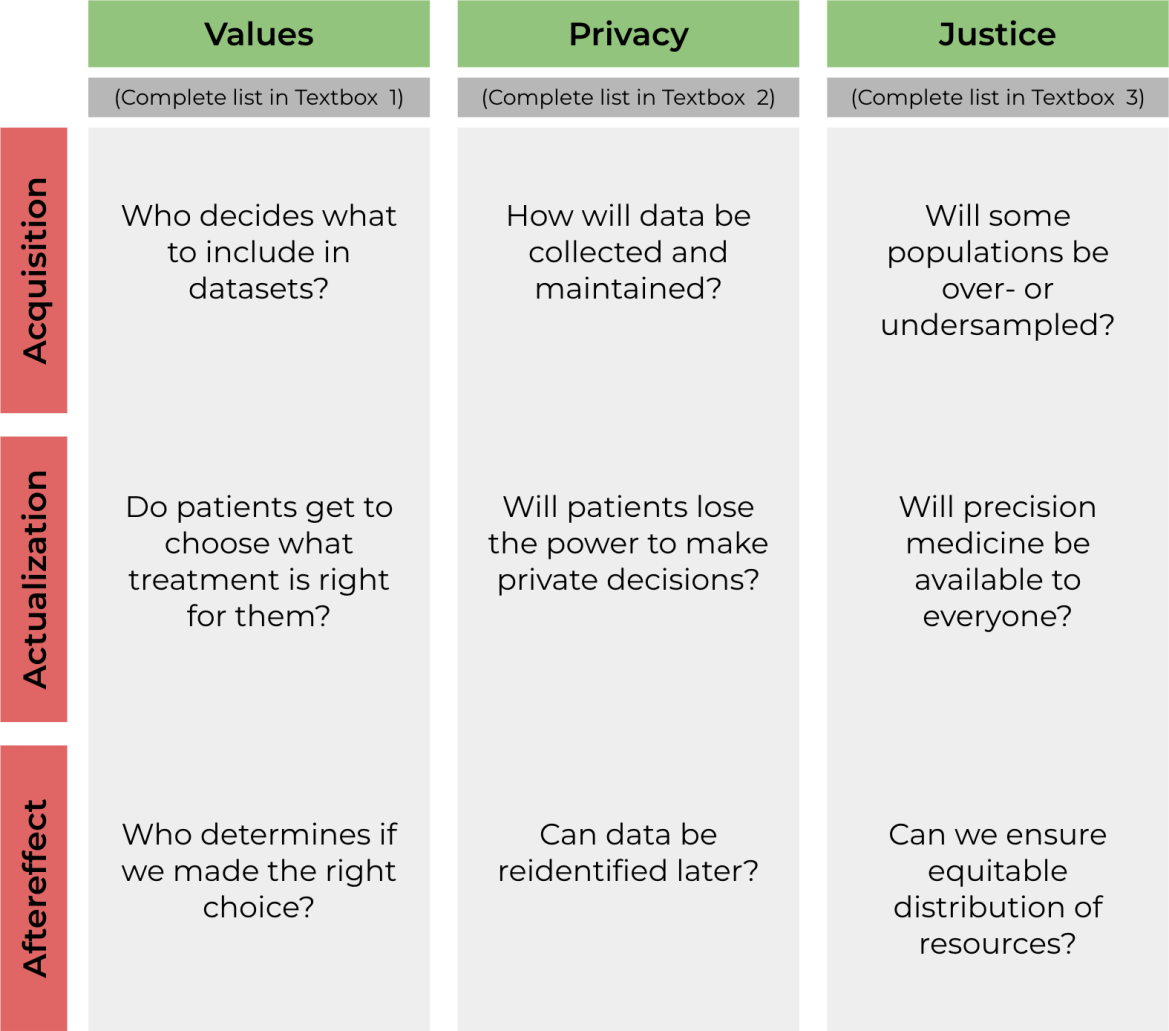
Priority matrix of ethical concerns for precision medicine.

In phase 4, participants grouped these challenges into common realms based on the nature of the ethical issue and where in the precision medicine process the issue was likely to occur. The participants identified 3 key time points: the acquisition of data, the actualization of precision medicine in practice, and the aftereffects of the process (Textboxes 1-3). The result of this mapping was a 3 by 3 matrix consisting of ethical challenges grouped into the domains of Values (eg, patient values and priorities as well as how precision medicine values those individuals), Privacy (eg, patient expectations as well as literal data security), and Justice (eg, overcoming and addressing systemic biases) as related to their temporal relationship to the precision medicine process (Figure 2).

Textbox 1. **Challenges identified by participants related to how the unique values of patients, providers, and the health care system will be incorporated into precision medicine.**

**Values**

- **Acquisition**

-  How are decisions made about which disease conditions to study and treat?
-  How accurate is data in the medical record at representing the whole of an individual?
-  How much does lifestyle matter and is it captured?
-  How will social determinants of health be evaluated?
-  Should individuals worry about the impact of nonmodifiable risk factors like family history or genetic predisposition?
-  What if individuals identify differently from measures used in precision medicine?
-  Can we mitigate the mistrust of people toward engaging with the system?

- **Actualization**

-  How do we address patient choice and preference in the face of precision recommendations?
-  What do we do if health care providers’ interpretations and values conflict with those of the patient?
-  How might this affect the patient-physician relationship?
-  Will patients be able to opt out?
-  Does the delivery of precision medicine change in the emergency scenario?
-  How do we ensure provider comfort with using precision medicine?
-  How do health care providers stay up to date with rapid changes in precision medicine?

- **Aftereffects**

-  How do we maintain humility as evidence for decision-making changes over time?
-  What if precision recommendations turn out to be wrong?
-  Might there be unintended psychological consequences like stress after decisions are made?
-  How do we ensure patient choice and preference is communicated longitudinally between medical teams?
-  What prevents a capitalist model from researching only the most common or lucrative conditions?

Textbox 2. **Challenges identified by participants regarding privacy in the precision medicine process.**

**Privacy**

- **Acquisition**

-  Who will be accountable for the collected information’s storage, maintenance, and safety?
-  How will protected information be handled?
-  How do we protect minors while ensuring appropriate data sharing with their caregivers?
-  How do we include underrepresented or excluded groups in a way that avoids perception of surveillance?

- **Actualization**

-  How will patients interface with their personal medical data?
-  Will patients lose the power to make private decisions about their own health when it may have a downstream effect on others?
-  How will changing evidence or incidental findings like new genetic risk factors be communicated with patients to ensure follow-up?

- **Aftereffects**

-  Can anonymized data be reidentified?
-  How do we ensure safe data sharing can occur among differing institutions or electronic medical record systems?
-  What are the legal ramifications of an infinitely variable standard of care?

Textbox 3. **Challenges identified by participants about how to ensure just care in the precision medicine model and process.**

**Justice**

- **Acquisition**

-  How do we incorporate all people into precision research?
-  Can we address disparities like access to care that make it hard for patients to participate?
-  Will those who are historically excluded continue to be so, while some populations will see over- and undersampling?
-  What do we do with historical data that may be biased?
-  Who will fund the research and data acquisition?
-  How are precision medicine priorities determined (ie, institutional level or government level)?
-  Could precision research resources be better spent addressing direct care access?

- **Actualization**

-  Will flawed data inputs result in flawed data outputs and recommendations?
-  How do we build a relationship of trust with a patient in the emergency department?
-  How do we prevent nonmodifiable predispositions from leading to biased care (ie, genetic determinism)?
-  How do we allow patient choice and values in the face of recommendations or limited resources?
-  Can variations in care still be equitable?
-  Will people get disproportionate care based on affordability?
-  How can we balance precision medicine safety with accessibility?

- **Aftereffects**

-  How will we maintain monitoring and vigilance of the accuracy of these systems?
-  How do we ensure systems are learning and adapting over time?
-  Can we ensure precision medicine is equitable across all patient populations?
-  Might the choice of who and what to study and treat have unintended consequences?
-  Might this further biased or racist ideologies and marginalize patients?

## Discussion

### Principal Findings

In this study, we have identified critical ethical challenges facing the implementation of precision medicine in the unique practice environment of the emergency department. The growing use of vast amounts of genomic, clinical, and lifestyle data to guide individualized therapies holds immense potential to improve emergency care. However, the “anyone, anywhere, anytime” mandate of emergency medicine presents distinct hurdles for the deployment of precision approaches that rely on the timely availability of complete patient information in a fast-paced and high-acuity setting. Using a modified nominal group technique, we engaged a diverse panel of emergency physicians to develop a consensus framework mapping key ethical challenges along the continuum of precision medicine implementation, from data acquisition to use in clinical practice to monitoring of downstream consequences. Crucially, this framework highlights the need to proactively identify and mitigate potential pitfalls to best realize the promise of precision medicine in the ED while promoting equitable access and benefit for the diverse patient populations we serve. Our findings provide a framework to guide research, practice innovation, and policy aimed at the ethical translation of precision medicine to the front lines of emergency care.

### Shared Ethical Challenges: Values, Privacy, and Justice

A recurring theme in our NGT discussion was “whose values are we accounting for?” [40]. Participants acknowledged the sometimes conflicting needs of the individual versus those of the system. Providers may recommend medications that prolong survival, but side effects may be undesirable or cost prohibitive for some patients [41]. The definition of high-quality care envisioned by precision medicine may not align with the individual goals of patients. There are multiple stakeholders driving the discourse of precision medicine—health care providers, health care insurers, regulators, and industry—the least empowered of which is the patient [40]. There were many questions raised regarding challenges to patient autonomy and the ambiguity of who is the ultimate decision maker in precision medicine and how that might change over time, as the needs of the many may be at odds with the needs of a few.

This tension maybe at odds with the ethical principle of autonomy [42]. The definition of respect for autonomy requires that patients are able to make their own decisions while incorporating their own values. This potentially extends to the use of their data and specimens, but the limits of patient autonomy remain under debate [43-45]. For example, the appropriate approach to consent for enrollment in biobanks that start in emergency departments remains under debate [46,47].

Ensuring that patient autonomy is maintained can be particularly challenging in the emergency care context. In the emergency department, providers make decisions with patients in a time-constrained setting, particularly compared with those historical clinical environments of precision medicine, such as oncology, which may occur over weeks. Not only does this limit time for discussion, it may also create a sense of external pressure to make a decision [48]. In such time-sensitive situations, patient personal information and preferences may be disclosed and decisions required under duress, thus also implicating privacy [49].

Privacy includes not only the protection of one’s identity and personal information, but also the ability to make choices without them being disclosed to others [50]. Another challenge to the patient’s privacy is that the practice of precision medicine in the emergency department will require massive amounts of data transiting among and across institutions and devices. Ensuring the safety and security of this protected information is a major hurdle for precision medicine initiatives, and the abovementioned time-sensitive nature of the emergency department encounter makes it necessary to share this data in real time, which is unique from other medical specialties [51-54]. In addition, when patients know that the information they provide to the system will be used to make recommendations for themselves or others, it may have downstream effects. For example, alcohol use and history for the allotment of liver transplant is one area that may leave patients in a moral quandary, unsure whether to be open with their behavior but fearing the effects of those disclosures [55,56]. Patient perceptions of the trustworthiness of health care providers and institutions will impact the success of any scientific or research endeavor, particularly among historically disadvantaged populations [57].

It is no surprise that our consensus framework includes justice as a key area for research in precision medicine ethics as it grows in the emergency department and in the prehospital setting [58-60]. Emergency physicians carry the distinct duty under federal law to provide care to anyone who presents for emergency care [61]. This duty is widely recognized to rest on a fundamental moral responsibility to provide care “promptly and expertly, without prejudice or partiality” [49]. At first it may seem that precision medicine would help to limit personal biases which might affect care, using data as opposed to gestalt or local practice variation. But structural biases may encode results and outcomes on patients that might inadvertently limit the benefits of the precision medicine model. When they are present in the initial development of the model, such as lack of representation and personal biases, affects the validity of results used to make targeted recommendations [62].

Justice, or more specifically with respect to medical ethics, distributive justice, emphasizes that resources are to be distributed fairly, equitably, and appropriately. This can be heavily hindered if providers rely on precision models to make recommendations without a critical appraisal of how well the results fit the patient in front of them. Even if done well, the outputs of precision medicine may not be available to all [63]. Precision medicine which is ultimately only accessible to a few but built on the data of many, would be an affront to the growing field of social emergency medicine [59]. In oncology, an area with a much longer history of precision medicine, 78% of patients and providers reported concerns regarding the accessibility of precision medicine [64].

### Continuum of Precision Emergency Medicine: Data Availability, Actualization, and Aftereffects

As noted by participants, challenges in implementing precision medicine in the emergency medicine context occur anywhere from when data is collected (or not collected) to the downstream effects of using precision medicine interventions. In emergency medicine, providers stand prepared to address a range of conditions at any time. We often must rely on incomplete patient histories (eg, due to the severity of illness or mental status changes frequently encountered in emergency medicine) and limited access to previous medical records (eg, our patients may be uninsured, underinsured, and without access to routine primary care providers) [65]. Precision medicine approaches will need to be deployable in a timely manner and maintain their precision with only the limited patient-specific information available to emergency physicians.

Furthermore, even when data are available, the actualization of precision medicine in the care environment may serve only to perpetuate systemic flaws in the health care system if that data is inaccurate, biased, or exclusionary from the start [7,66]. A 12-year review of the National Hospital Ambulatory Medical Care Survey (NHAMCS) illustrates this risk: Black patients were 10% less likely to receive immediate or emergent Emergency Severity Index (ESI) scores and to be admitted, but 1.26 times more likely to die in the emergency department or the hospital than White patients [65]. Using such historically biased data to make precision medicine recommendations about resource allocation could amplify these disparities. While precision medicine aims to deliver individually tailored care, it still requires categorizing patients into discrete groups by shared characteristics—a process that carries its own implications and potential for bias [4,67].

Additional logistic concerns for the actualization of precision medicine in emergency departments, include costs, legal issues, and patient education. Efforts made to collate data from emergency care settings to be used in precision medicine research may result in products or treatment pathways unaffordable to these patients [68-70]. This may be mitigated through insurers [71], but the emergency department is a safety net, and as such this approach may not benefit the most vulnerable patients while those who are insured may find that their plans have little incentive to offer this option [72]. Research has shown that the cost of precision medicine is a concern shared by patients and providers alike [63,64].

Even when payment is not an issue, a data- and machine-supported approach may not be conducive to truly informed consent given the limitations of adequately educating emergency department patients regarding how precision medicine and its associated research works [63,73]. Beyond the challenge of consenting in the emergency context, providers must also consider issues regarding disclosing incidental information and the potential changes associated with the use of data over time which interconnected to other growing datasets. Stewardship of these data, defined as “the responsible management of something entrusted to one’s care” [74], will be critical to maintaining trust of patients and communities as this science evolves [57].

Discussion in this NGT process highlighted the need to actively maintain a holistic view of the patient without being overly reductive to these categories. Currently, only a limited amount of data is collected with respect to health measures or lab studies, and even less is recorded for social determinants of health. Future work should focus on systematic collection of environmental and social factors in emergency care settings, including neighborhood-level data on environmental exposures, community resources, and barriers to care access. However, this data collection must be done thoughtfully to avoid reinforcing biases—for example, by ensuring community engagement in data collection methods, using validated measurement tools that have been tested across diverse populations, and analyzing social factors as modifiable system-level variables rather than immutable patient characteristics. In emergency medicine specifically, this could include developing standardized screening tools for social needs that are feasible in acute care settings, creating automated systems to gather community-level data that do not burden individual patient encounters, and establishing partnerships with community organizations to better understand local health contexts. These approaches could help create more comprehensive precision medicine models while maintaining emergency medicine’s core mission of providing equitable care to all patients.

### Limitations

The use of a NGT and participant diversity are key strengths of this study. However, several limitations warrant discussion. While NGT aims to limit the biases associated with other consensus models, expertise bias is still a potential limitation, as all participants were aware of and knowledgeable on this topic beforehand. While this expertise strengthens the confidence in the working knowledge of the topic, it can also lead to anchoring. We took several steps to limit its impact. NGT, by way of its structured format, is meant to limit the influence of any one dominant individual in discussion [39]. In addition, we included a diverse spectrum of emergency physicians from residents to attending physicians with varying years of experience, as well as emergency physicians with a variety of subspeciality training.

Self-selection bias should also be considered, as participants volunteered in response to an email invitation to the Society of Academic Medicine Ethics Committee listserve. This recruitment method may have attracted participants with stronger interest in ethics and precision medicine, though their engagement likely enhanced the depth of insights generated during the NGT session.

Finally, though the number of participants was within ideal range for an NGT [24], the relatively small group size remains a limitation. Future research could benefit from additional strategies that would incorporate a higher number of emergency physicians and experts from outside of the field which might offer perspective to our work.

### Conclusions

Realizing the promise of precision medicine’s “right person, right place, right time” must be balanced by the context of the “anyone, anywhere, anytime” of emergency medicine. Our work has illuminated a range of concerns for the development of precision emergency medicine, and provides a focused framework for the most pressing ethical considerations along the continuum of data acquisition to implementation. This framework may be used to direct research and innovation toward addressing these challenges in the emergency medicine setting.

## Supplementary material

10.2196/68371 Multimedia Appendix 1 Additional tables.

## Abbreviations

CHD1: chromodomain helicase DNA binding protein 1
COREQ: Consolidated Criteria for Reporting Qualitative Research
CYP: cytochrome P450
ESI: Emergency Severity Index
NGT: nominal group technique
NHAMCS: National Hospital Ambulatory Medical Care Survey
