# Supplementary material for: Identifying High-Priority Ethical Challenges for Precision Emergency Medicine: Nominal Group Study
Source: JMIR Form Res. 2025 Feb 6;9:e68371. doi: 10.2196/68371 (PMC11825900; doi:10.2196/68371)
Supplement: Multimedia Appendix 1 [file formative-v9-e68371-s001.docx]

Table S1: Nominal Group participant specialty and level of training.

| Initials | Specialty | Level of Training | Practice Setting |
| --- | --- | --- | --- |
| AF | Emergency, Pediatrics | Attending, Early | Academic Center, County Hospital |
| TD | Emergency, Education | Attending, Early | Academic Center, Community Hospital |
| DW | Emergency, Pediatrics, Education | Attending, Mid | Academic Center, County Hospital |
| DH | Emergency | Resident | Academic Center, County Hospital, Integrated Managed Care |
| SC | Emergency | Resident | Academic Center, County Hospital, Integrated Managed Care |
| DD | Emergency, Informatics | Fellow | Academic Center, Veterans Affairs (VA) |
| EI | Emergency, Ethics | Attending, Late | Academic Center, County Hospital |
| LS | Emergency, Education | Attending, Early | Academic Center, Integrated Managed Care |
| NP | Emergency | Resident | Academic Center, County Hospital, Integrated Managed Care |
| SM | Emergency | Resident | Academic Center, County Hospital, Integrated Managed Care |
| GT | Emergency | Attending, Early | Community Hospital |
| MD | Emergency | Resident | Academic Center, County Hospital, Integrated Managed Care |

Table S2: Complete list of questions generated by participants in steps one and two.

| **Participant 1** |
| --- |
| Complicating payer structures (things getting more expense without getting anything out of it) |
| Differential treatment across institutions |
| Things getting more expensive |
| Proper systems in place for error reporting and monitoring |
| Weight issues across institutions ([Institution X] does genetics, so [their] precision is going to be about genetics, vs. another institution might have a tox focus, etc.) |
| Privacy concerns when cohorting patients - who is similar and who is not? determining that |
| Provider comfort with deployment of differential treatment strategies |
| Difficulty in ensuring equitable across multiple groups |
| **Participant 2** |
| Family history / genetic predisposition concerns* - example opiates |
| Inability to pay (offer to a patient or not, if you know they can't pay) |
| **Participant 3** |
| What prevents companies from researching the most common (lucrative) condition and focusing on this |
| Where is the border between privacy and less private for the benefit of hoping interventions will be tailored to you |
| Is practicing different medicine on different patients fair? just? what language do we use here |
| How to navigate between deviant phenotypes and societal/envt barriers that disable people |
| How to incorporate everyone into precision research (barriers to informed consent) |
| Are people just going to share their data to be unwillingly fixed |
| Are you losing the power to make decisions about how to manage your own differences |
| How to capture social determinants of health? Do we capture them? |
| How accurate is the medical record in representing entities? |
| **Participant 4** |
| Difficulties remaining current |
| Providers anchoring on past determinations (correctly or incorrectly) |
| A number of disparities that would make it hard to participate (financial, housing, recent immigrants, without PCP) |
| Lacking information about how genetics change over time? |
| Issues transferring information between health systems and health record systems |
| How much does lifestyle matter? |
| Issues incidental findings and follow-up |
| Do we have a lens of prevention vs just acute medical conditions? |
| Who is going to fund the research (response to opiates* example) - historical studies |
| **Participant 5** |
| What are we going to do around minors, is this going to be shared with guardians |
| Flawed data going in will lead to flawed data going out |
| Data sharing among institutions EMRs that speak to each other |
| Who will pay? individual vs societal - extreme cost of care already |
| How are we measuring precision medicine from a quality lens (how do we know we are doing it right/well) |
| **Participant 6** |
| If we think social determinants of health are inputs, are patient values inputs? |
| Privacy is a concern |
| **Participant 7** |
| Oversampling some populations and undersampling others |
| Opportunity further racist ideology and marginalize patients - race is genetic and biologic is one argument in our country |
| Will precision-medicine be the same for diagnoses and populations |
| Expensive |
| How to decide what disease conditions to study and treat? |
| Will who we decide to study and treat have unintended consequences? |
| What if we are wrong (precision medicine is wrong)? |
| Reducing our patients to a few factors and not holistic human -- harmful to patient and doctor |
| How are we going to teach it? |
| Will all programs have access to a person who can teach this? |
| What about getting the right treatment for patients is not right for that human - "recommendation of admission". Can precision medicine pivot? if not inline with patients |
| Will non-precision medicine be available to patients or do they have to do precision-medicine pathway |
| **Participant 8** |
| How will genetic info be collected, stored, maintained? who will be accountability to this |
| Who will be accountable for the collected information |
| Provider- do we know how to interpret this information and does that change in an emergent scenario |
| Does how we interpret PEM change in an emergent scenario |
| Equity - how do we include underrepresented or excluded groups in a way that avoids surveillance |
| What will corporate influence in this area be? |
| Can different care still be equitable "separate but equal" in outcomes? |
| Can perception of different care still be equitable |
| How do we maintain humility as we learn more about genetics and epigenetics? |
| **Participant 9** |
| Should we be investing in PEM when many have no access to care |
| Will insurance companies access this info and use it against you |
| Unintended psychological consequences (person knows they will get a disease, lives in fear) |
| Mistrust of people/system |
| Parents consent on behalf of their child and they never wanted to know? |
| Potential lawsuits |
| **Participant 10** |
| Patient interface with own medical information and ability to interpret and apply this information |
| Gender issues? identity issues? |
| How do we ascribe value to different educational choices and the ways they impact their health care |
| The discoverability and legal proceeding have more information and how that impacts insurance/legality |
| Who owns this data? |
| How can we make it safe while also making it accessible |
| Accuracy in obtaining demographic information (is patient listing this or someone else assigning it) |
| **Participant 11** |
| How do you make sure goals of precision medicine are not being changed when going from hospital—>provider—>patient |
| How do we keep the interest of patient first |
| How do we present ideas in a way that everyone can understand? |
| Feedback. How do we make systems adaptable over time |
| How do we keep patient’s choices in precision-medicine |
| How do we keep the personalization in precision medicine |
| Social determinants: gender, financial, socially. How do we communicate this? |
| **Participant 12** |
| How do we make judgements on what is most important to prioritize |
| Doctors are humans too and may have judgements and values that may conflict with patients |
| Trust generally requires relationship built over time and how do we do that in the ED |
| Feedback from EM to PCP and vice versa |
| Genetic risk factors vs modifiable or behavioral risk factors and how to play in care |
| Through patient education how can we strategize to engage historically hesitant groups from participation? |
